# On the scaling properties of oscillatory modes with balanced energy

**DOI:** 10.3389/fnetp.2022.974373

**Published:** 2022-11-08

**Authors:** Dobromir G Dotov

**Affiliations:** LIVELab, Psychology, Neuroscience and Behaviour, McMaster University, Hamilton, ON, Canada

**Keywords:** 1/f, EEG, homeokinetics, homeostasis, physiological control, scaling, aperiodic, oscillations

## Abstract

Animal bodies maintain themselves with the help of networks of physiological processes operating over a wide range of timescales. Many physiological signals are characterized by 1/*f* scaling where the amplitude is inversely proportional to frequency, presumably reflecting the multi-scale nature of the underlying network. Although there are many general theories of such scaling, it is less clear how they are grounded on the specific constraints faced by biological systems. To help understand the nature of this phenomenon, we propose to pay attention not only to the geometry of scaling processes but also to their energy. The first key assumption is that physiological action modes constitute thermodynamic work cycles. This is formalized in terms of a theoretically defined oscillator with dissipation and energy-pumping terms. The second assumption is that the energy levels of the physiological action modes are balanced on average to enable flexible switching among them. These ideas were addressed with a modelling study. An ensemble of dissipative oscillators exhibited inverse scaling of amplitude and frequency when the individual oscillators’ energies are held equal. Furthermore, such ensembles behaved like the Weierstrass function and reproduced the scaling phenomenon. Finally, the question is raised whether this kind of constraint applies both to broadband aperiodic signals and periodic, narrow-band oscillations such as those found in electrical cortical activity.

## 1 Introduction

Neural and cognitive function distributes its activity across a wide range of temporal and spatial scales replete with feedback loops and noise (Buzsaki and Draguhn, 2004). There is renewed interest in the scaling properties of aperiodic ensemble activity of neural systems and the potential role of critical dynamic regimes (Sporns, 2022). Neural function is also enmeshed with other faster and slower processes of autonomous physiological control. Network physiology studies such nonlinear control across scales and the fluid reorganization between distinct network modules and dynamic motifs (Ivanov, 2021). One way to quantitatively address such networks would be to try to decompose them into individual units and their interactions, which can be described as a micro-to-macro approach. One can also seek theoretically-motivated meso- and macro-variables that offer insight into the underlying complexity. For example, quantifying the variability and distributional properties of a collection of physiological time-series can help distinguish between homeostatically regulated target dimensions and dynamic response dimensions that absorb perturbations (Fossion et al., 2018), in line with Ashby’s theoretical search for *essential variables* (Ashby, 1960). Another possibility is to analyze and interpret the observed inverse scaling relation between frequency and power, in its most general form known as 1/*f* noise, as it has been mapped to optimal and healthy ranges of performance (Lipsitz and Goldberger, 1992; Stergiou et al., 2006).

The phenomenon of 1/*f* noise is observed in physical (Bak, 1999), neural (Buzsáki, 2009), and cognitive systems (Gilden et al., 1995; Kello et al., 2007). Zipf’s law is a similar function, observed universally in human languages, that consists of inverse scaling between word-use frequency and its rank. Beginning with Mandelbrot’s proposal (Mandelbrot, 1953), there has been a tradition of explaining the ubiquity of such inverse scaling in terms of maximizing information-theoretic quantities in multi-scale systems (Zhang, 1991; Costa et al., 2002; Yu et al., 2005; West et al., 2008). For example, the Zipfian scaling is consistent with an optimal trade-off between the use of few words often to minimize effort and the use of rare words to maximize communication (Zipf, 1949; i Cancho and Solé, 2003).

In search for organizing principles for multi-scale network physiology, we borrow from Arthur Iberall’s *homeokinetics*, a set of ideas about homeostatic regulation in thermodynamically open complex systems. Biological systems distribute their internal regulation among multiple scales and operational modes of activity. . Fluxes and potentials of metabolic energy are involved at all levels and stages of operation; at every scale of description, our bodies use energy and produce heat to do work (Iberall and Soodak, 1987). These can be seen as limit-cycles because of the rough periodicity and relative resistance to perturbation. Importantly, switching among such modes is most efficient if they are energetically unbiased. As raising either the amplitude or frequency of a real physical system takes energy, to be unbiased, physiological action modes must exhibit a trade-off between frequency and amplitude in the form of the well-known 1/*f* scaling, (Iberall and Soodak, 1987; Iberall, 1995). This is a thermodynamic counterpart to the information-theoretic arguments. Here we test this idea by investigating the properties of a model that consists of an ensemble of oscillatory dissipative units with a spectrum of intrinsic frequencies but constrained by their energy levels.

## 2 Material and methods

### 2.1 An ensemble of canonical-dissipative oscillators as a Weierstrass function

The model consists of superimposed independent processes (Eliazar and Klafter, 2009). The individual units are not stochastic signals, however, but oscillators with a physically-interpretable energy parameter. The unit of the system is the canonical-dissipative oscillator that consists of a conservative part with a frequency parameter *ω*, a velocity-dependent dissipative part, and noise term with parameter *Q* (Haken, 1973; Schweitzer et al., 2001; Frank, 2010; Mongkolsakulvong and Frank, 2010; Frank et al., 2011).

$$\begin{array}{c} \ddot{x}=-\omega^{2}x-\gamma \dot{x}\left(H-b\right)+\sqrt{Q}\Gamma \left(t\right) \end{array}$$ (1)

The energy of the oscillator is given by the following equation.

$$\begin{array}{c} H=\frac{\omega^{2}x^{2}}{2}+\frac{{\dot{x}}^{2}}{2} \end{array}$$ (2)

This is in analogy with Hamiltonian mechanics which aims to express oscillatory dynamical systems not in kinematic coordinates such as position but in a coordinate system of potential and kinetic energy. The energy given by Eq. 2 is balanced by a pumping parameter *b*. Due to the system’s dissipative nature, in time *H* converges to the pumping parameter *b* (Frank, 2010), and *γ* determines the speed of this convergence. Interestingly, even though the initial motivation was different, Eqs (1) and (2) together amount to the same form as the so-called hybrid Van der Pol–Rayleigh (Kay et al., 1987) oscillator, plus an added stochastic term. The hybrid oscillator was a phenomenological model that accounted for important characteristics of human rhythmic movement, one of them being an inverse relation between movement amplitude and frequency (Haken et al., 1985; Kay et al., 1987). Note also that equations of the same form have been introduced elsewhere as an ‘energy oscillator’ (Buchli et al., 2006).

To complete the model, we defined an ensemble of *N* = 10 canonical-dissipative oscillators to be solved numerically, where dot-notation indicates the time-derivative.

$$\begin{array}{c} {\ddot{x}}_{j}=-\omega_{j}^{2}x_{j}-\gamma {\dot{x}}_{j}\left(H_{j}-b\right)+\sqrt{Q}\Gamma_{j}\\ H_{j}=\frac{\omega_{j}^{2}x_{j}^{2}}{2}+\frac{{\dot{x}}_{j}^{2}}{2} \end{array}$$ (3)

The ensemble at time point *i* was the sum across the state variables *x*_*j*_ of the *N* oscillators.

$$\begin{array}{c} y_{i}=\Sigma_{j=1}^{N}x_{ji} \end{array}$$ (4)

**FIGURE 1:**
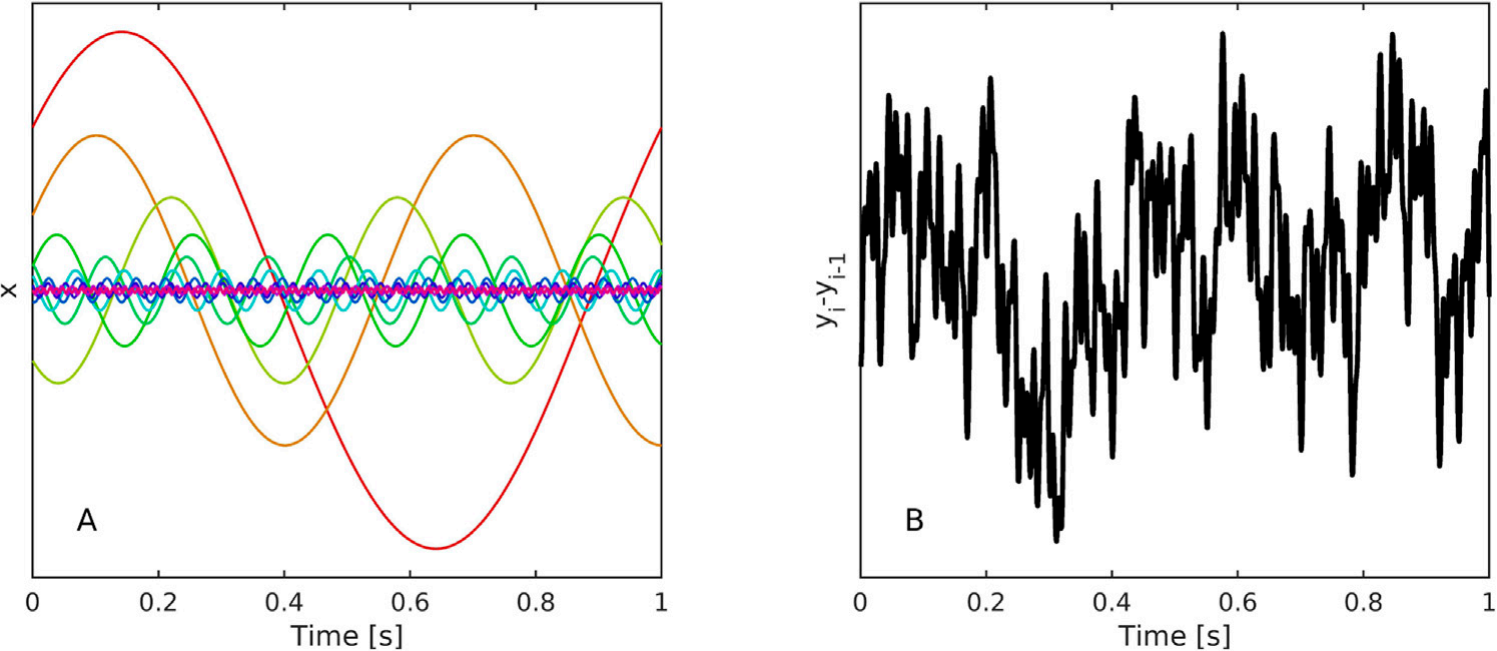
**(A)** Representative trial involving ten oscillators simulated using Eq. 3, 4 in the condition of constant energy *b* across oscillators. **(B)** Their differenced ensemble summed activity, Eq. 4, has a scaling exponent of *α* = .9703.

This reduces the *N* harmonic waves see Figure 1A to a one-dimensional time series, the increments of which exhibit scaling properties, see Figure 1B.

Importantly, this model works like a Weierstrass function which also is a summation of harmonic waves with an inverse scaling relation between amplitude and frequency.

$$\begin{array}{c} W_{\alpha }\left(x\right)=\overset{\infty }{\underset{n=0}{\sum }}b^{-n\alpha }\cos\left(b^{n}x\right) \end{array}$$ (5)

The Weierstrass function helped spur the study of fractal objects by introducing a benchmark example of a function that is continuous in time yet everywhere-singular for certain parameters (Mandelbrot, 1982). Its *α* parameter agrees well with its Hurst exponent *h* (Zhao et al., 2013). In the same vein, both Eq. 5 and the model in Eqs. 3, 4 are special cases of a more general approach that generates Lévy laws and 1/*f* ^*β*^ noises using random frequencies, random amplitudes, and any seed pattern *in lieu* of harmonic waves (Eliazar and Klafter, 2009). Note that the fractal properties of the Weierstrass function depend on its parameters. Similarly, the outcome of the present approach depends on model and numerical integration parameters such as *dt*, *N*, *Q*, and the range of *ω*. We used hand-picked parameters as it is beyond the scope of the present work to study the full parameter space. Eqs. 3 and 4 should be seen as a conceptual model of how a potentially meaningful variable could constrain oscillatory physiologically processes, not as a robust generative model of physiological signals.

### 2.2 Simulations

We simulated trials by integrating the system and taking its summed ensemble, Eqs. 3 and 4 with an Euler step of *dt* = 10^–3^, random initial conditions, and a trial duration of 1 s plus an initial transient part which was discarded. Each oscillator had a different angular frequency, *ω*_*j*_ = 2 *π* 1.6681^*j*−1^ rad*s^−1^, *j*={1,2,...,10}, resulting in a range from 1 to 100 *Hz*. The other two parameters were less relevant to the present question and were identical across units, *γ* = 10 and *Q* = .01. *γ* determines how quickly each oscillator settles on its limit cycle and would play a more prominent role if transient dynamics and effects of different initial conditions were to be investigated. *Q* scales the magnitude of an additive Gaussian force, Γ∼N(0,1) . To simplify the analysis, *Q* was chosen to be negligible. The initial phases were random and the oscillators were not coupled, in line with the assumption that physiological processes need a degree of independence to maintain their function (Iberall, 1995). They were constrained, however, by an energy resource *b*_*j*_.

In addition to the primary scenario defined by equal energy per unit, we investigated whether scaling properties depended on the distribution of the energy pumping parameter. We simulated ten trials in each of four different conditions. The first three conditions were characterized by a pumping parameter that was either monotonically *increasing*, *constant*, or monotonically *decreasing* with respect to the intrinsic frequencies of the oscillators. For the increasing condition, *b* was given by *b*_*j*_ = 2.5^.8*j*+2^. The decreasing condition was the reverse of that. In the constant condition, all *b*_*j*_ = 10^2^. In the fourth condition, the parameter was *selective*, whereby one oscillator was given privileged access to a larger pumping term, *b*_*j*=5_ = 10^3^ and *b*_*j*≠5_ = 10^1^. At present, we do not address switching and coordination among modes. Future work can explore the role of competitive coupling to the energy resource.

## 3 Results

**FIGURE 2:**
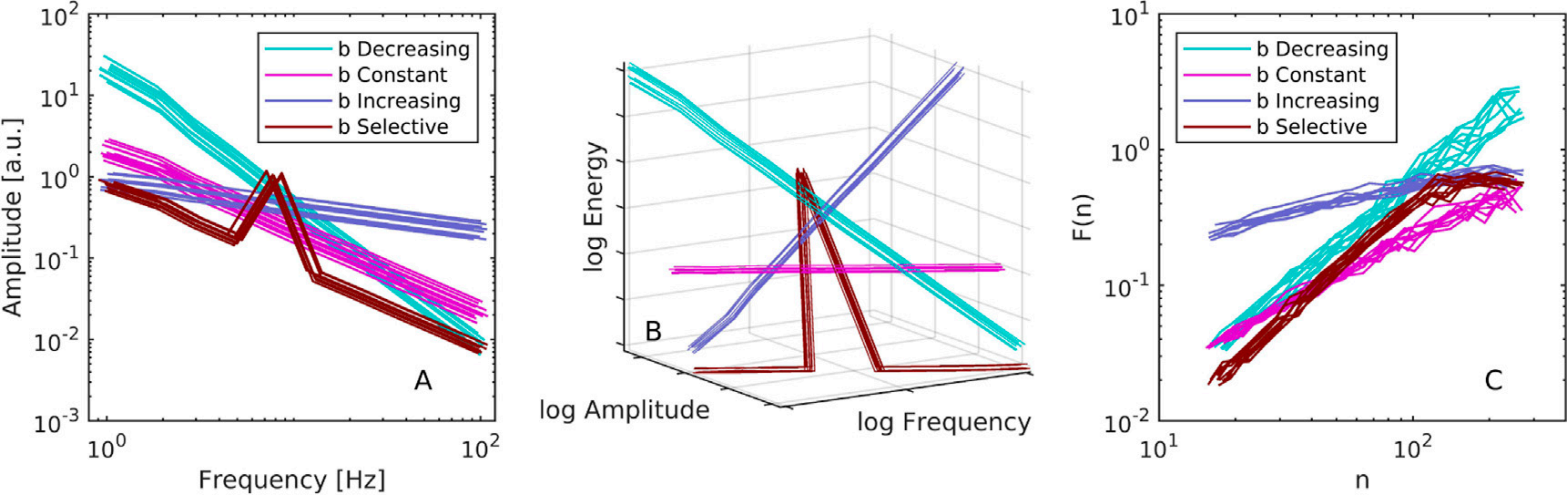
**(A)** The relationship between amplitude and frequency of the individual canonical-dissipative oscillators in Eq. 3. Each of the ten lines in a given condition corresponds to a simulated trial run comprising *N* =10 parallel oscillators, each with a different intrinsic frequency in the range from 1 to 100 Hz. Conditions are color-coded and refer to the distribution of the pumping parameter *b* relative to the oscillators’ frequencies. Specifically, *b* could increase, decrease, stay constant, or stay constant and low with the exception of one selected privileged frequency. Lines were jittered for visibility. **(B)** The same figure as **(A)** but with an added axis for the oscillators’ energies reveals that, according to the present definition of oscillator energy in Eq. 2, 1/*f* scaling is associated with a flat energy spectrum. **(C)** *DFA* analysis of the ensemble-summed time-series. The fluctuation functions confirm that constant pumping, or equal energy across the component oscillators, results in scaling exponent approximately equal to unity.

First, we verified that the energies of the individual oscillators in Eq. 3 responded to the pumping parameter *b*. We pooled all units from all trials (*n* = 400 from ten units in ten trials in four conditions). The input parameters *b* was regressed against the time-average of the observed energies *H* defined by Eq. 2. As expected, the agreement was strong with *R* ^2^ = .9999 and a slope of 1.0054. Next, we analyzed the scaling of oscillator amplitudes. In each trial consisting of *N* = 10 parallel oscillators, we regressed linearly in log-log space their observed trial-averaged half-amplitudes with respect to their frequencies, see Figure 2A. As expected, the scaling was inverse for constant *b* (a mean *M* = −1.016 and standard deviation SD  = 0.001 across the ten simulation runs). This serves to confirm that the model defined by Eq. 3 effectively mimics one aspect of the Weierstrass function. The exponent was steeper for decreasing *b*, that is with more energy at the low-frequency oscillators (*M* = −1.724, SD  = 0.012). Conversely, with an increasing *b* which put more energy in the higher-frequency oscillators, the scaling was less steep (*M* = −0.310, SD  = 0.009). In the selective condition, the regression yielded inverse scaling (*M* = −1.045, SD  = 0.002), consistent with the fact that nine out of ten oscillators had equal energy. Importantly, Figure 2A also shows that the one privileged oscillator produced a prominent peak.

Having computed the observed individual unit energies using Eq. 2, we looked at their relation to amplitude and frequency. As implied by the strong agreement between trial parameters *b* and the observed energies, the energy profile was flat with respect to the amplitude and frequency spectrum only in the equal energy condition, see Figure 2B.

Next, we analyzed the scaling properties of the ensemble *y*_*i*_ defined by Eq. 4. We used detrended fluctuation analysis, a method for the scaling exponent of self-affine signals that has become one of the benchmark tools in the analysis of physiological time-series (Peng et al., 1994; Buldyrev et al., 1995). It gained prominence with its application to inter-beat intervals in cardiac recordings (Peng et al., 1995). It relies on the fact that aperiodic signals with scaling properties also exhibit scaling of the amount of fluctuation, as quantified by the mean of running windows of root-mean-square, relative to the size of the windows, see Figure 2C. The analysis parameters consisted of first-order detrending, a minimum window size of ten points, and no integration of the input data because the increments of the summed ensemble exhibited scaling. As Figure 2C shows, in the constant *b* condition *y*_*i*_ was a time-series with a scaling exponent that approximated ideal *α* = 1 (*M* = 0.972, SD  = 0.035 across the ten simulation runs). Favouring higher frequencies with an energy parameter that increased with frequency reduced the exponent (*M* = 0.398, SD  = 0.007). Favouring the lower frequencies increased the exponent (*M* = 1.551, SD  = 0.096). Having a mix of equal energy plus one emphasized frequency in the selective *b* condition also increased the exponent (*M* = 1.282, SD  = 0.008). Note that scaling analysis in the spectral domain is also motivated in the present scenario, although the results would depend on how the frequency bins are aligned with the frequencies of the component oscillators. This implies that our method cannot be considered an ideal generative model for 1/*f* noise, at least not until the role of more dense sets of component oscillators is investigated.

The present ideas suggest the possibility that isolated narrow-band modes would exhibit the same association between amplitude and frequency. The scaling of individual physiological oscillatory processes has not been investigated as much from this perspective, aside from the observed inverse relationship between amplitude and frequency in repetitive movements (Haken et al., 1985; Kay et al., 1987). To this end, here we propose a brief re-analysis of published summaries of narrow-band ranges of electrical cortical activity (Stern, 2013; Reilly, 2015).

**FIGURE 3:**
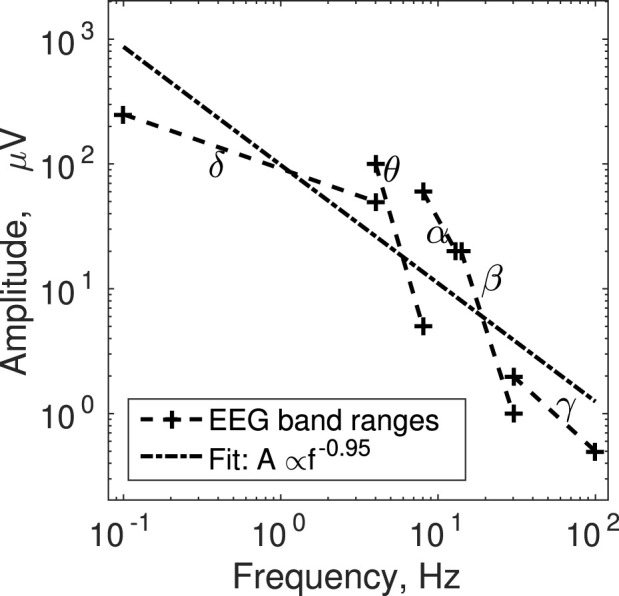
The ranges of narrow-band electrical cortical activity are shown in terms of their respective upper left (maximum amplitude, minimum frequency) and lower right (minimum amplitude, maximum frequency) corners. The power-law fit through these data is also shown.

Cortical activity as recorded on the surface of the scalp with EEG can have pronounced narrow bands that reflect the conscious state and ongoing cognitive activity of the participant (Stern, 2013; Reilly, 2015). To name a few examples, deep sleep is associated with increased activity in the *δ* band (.1–4 Hz), drowsiness with the *θ* band (4–8 Hz), awake but mentally relaxed state with *α* (8–13 Hz), focusing and mental agitation with *β* (14–30 Hz), and multi-sensory stimulation and integration with *γ* (30 + Hz). We pooled together the EEG amplitude and frequency ranges per band reported in sources (Stern, 2013; Reilly, 2015). We selected these sources for convenience because they offered representative summaries of standard EEG bands, not datasets of raw EEG recordings. We fitted a power-law to the data points characterizing the boundaries of the bands. Because there were some discrepancies between the two sources, we used the extreme values, namely maximum amplitude at minimum frequency and minimum amplitude at maximum frequency. As Figure 3 shows, the overall trend is close to inverse scaling of amplitude with frequency, *A* ∝ *f* ^−.95^. This question requires more thorough dedicated investigation in the future. To confirm this observation, the amplitude of focal EEG oscillatory activity needs to be dissociated from the underlying broadband 1/*f* profile of EEG (Donoghue et al., 2020).

## 4 Discussion

Here we presented a modelling study that investigated a possible link between 1/*f* scaling of broadband physiological signals and the energy that constrains the underlying physiological control modes. We discovered that the constraint on energy led our model system to mimic the Weierstrass function. This is interesting because the latter is a paramount example of a fractal object but, to our knowledge, it does not have a physical grounding, originally being known as a so-called mathematical monster (Mandelbrot, 1953). Similarly, the canonical-dissipative oscillator was not designed for the present purposes, we merely put a small number of units in an ensemble characterized by a broad frequency spectrum and applied a theoretically-motivated constraint on their energy parameter. The canonical-dissipative oscillator is defined by an intrinsic frequency, a dissipative part, and energy-pumping which is balanced by the dissipation, allowing the system to settle on a limit-cycle with an energy-dependent amplitude. (Haken, 1973; Frank, 2010). This was motivated by ideas from Arthur Iberall’s theory of complex systems (Soodak and Iberall, 1978; Iberall and Soodak, 1987). According to Iberall, physiological signals reflect multiple action modes existing on a broad spectrum of time- and space-scales and these modes tend to have equal energies. The equal-energy condition ensures that switching among them is unbiased and catalytic, meaning that switching among modes is at a considerably lower energy level than the modes themselves.

Understanding the role of catalytic processes in the self-organization of biological function was advanced further by the notion of autocatalytic sets (Kauffman, 1993; Kauffman, 1995). This is related to a popular idea about scaling phenomena, namely that they reflect a system poised in a critical state where stability and adaptability are balanced optimally. This is relevant to neural dynamics as well, given renewed interest in critical phenomena in the brain (Sporns, 2022). We are yet to determine how the idea of energy constraint fits within the existing landscape of complex systems theories. For example, the original sand-pile model employed a damped pendulum as a physical metaphor for the constitutive unit of the system (Bak et al., 1988), hence an analysis in terms of potential and kinetic energy must be possible in principle. More recently, a novel notion of complex system stability has gone beyond static homeostasis. Systems with *antifragility* not only maintain the stability of a target internal variable when exposed to a perturbation but also grow and increase their capacity to sustain future perturbations (Taleb, 2012). The connections between criticality and antifragility are only beginning to be explored computationally (Pineda et al., 2019).

The idea that a physical system needs more energy to increase its amplitude and/or frequency is a simple one, yet its relevance to neural oscillations is yet to be determined. There is a related observation, however, an established time-mass relation in neural electrophysiology: the magnitude and period of a wave tend to be associated with the size of the underlying substrate of co-activated neurons (Buzsaki and Draguhn, 2004; Grigolini et al., 2009; Aru et al., 2015). It is important to point out that the approach advanced in the present work does not fit easily with current thinking about the nature of aperiodic and oscillatory neural dynamics. These two kinds of dynamics tend to be associated with different generative mechanisms (He, 2014), whereas our assumption was that they both obey the same condition of balancing energy. We adhere to the idea that physical constraints on function and structure are simpler and more fundamental than biological mechanisms and it is not impossible that different biological mechanisms are subject to the same constraint.

It is an important reminder that power laws rarely provide an ideal fit of empirical scaling phenomena in EEG (Bedard et al., 2006) and generally in biological systems (Clauset et al., 2009). Still, the overall trend is a fact and its source needs to be identified. Indeed, biological structure and function often can be seen as a combination of a global constraint based on physical law and local constrains based on specific adaptations (West and Goldberg, 1987). In addition to developing generative models of self-organizing systems with scaling properties, it is important to understand the various constraints that shape the evolution and development of physiological networks.

## Data Availability

The original contributions presented in the study are included in the article/supplementary material, further inquiries can be directed to the corresponding author.
